# The cytocam video microscope. a new method for visualising the microcirculation using incident dark field (IDF) technology

**DOI:** 10.1186/2197-425X-3-S1-A601

**Published:** 2015-10-01

**Authors:** SD Hutchings, J Wendon, S Watts, E Kirkman

**Affiliations:** Biophysics Division, Defence Science and Technology Laboratory, Salisbury, United Kingdom; Critical Care, Kings College Hospital, London, United Kingdom

## Introduction

Imaging the microcirculation provides valuable information regarding perfusion in a range of shock states. We report a new microcirculatory assessment device, the Braedius Cytocam, an Incident Dark Field (IDF) video microscope, and compare it with the most commonly used precursor device, the Microvision Microscan which utilises side stream dark field (SDF) imaging.

## Methods

This comparison study was performed within an existing experimental model of traumatic hemorrhagic shock. The study was approved under the Animals (Scientific Procedures) Act 1986. Six terminally anaesthetized pigs were subjected to injury and haemorrhage followed by resuscitation with a variety of fluids. Microcirculatory measurements were taken from the sublingual region at baseline (pre-injury and haemorrhage) and in the thirty minute shock phase following controlled haemorrhage. Measurements were taken using the SDF device immediately followed by the IDF device. At least three, and ideally five, measurements were taken using each device. Video sequences were anonymised and then exported for analysis using a semi automated software tool (Automated Vascular Analysis v 3.0, Microvision Medical). Videos were analysed for Total Vessel Density (TVD) and Microvascular Flow Index (MFI). In order to assess image quality, identical images of the sublingual microcirculation of one of the investigators was taken using the two devices. Image contrast was assessed using Image J software, a pixel assessment tool. Differences between groups was assessed using paired t tests or Wilcoxon matched - pairs signed rank tests depending on normality of data.

## Results

**Figure 1:**
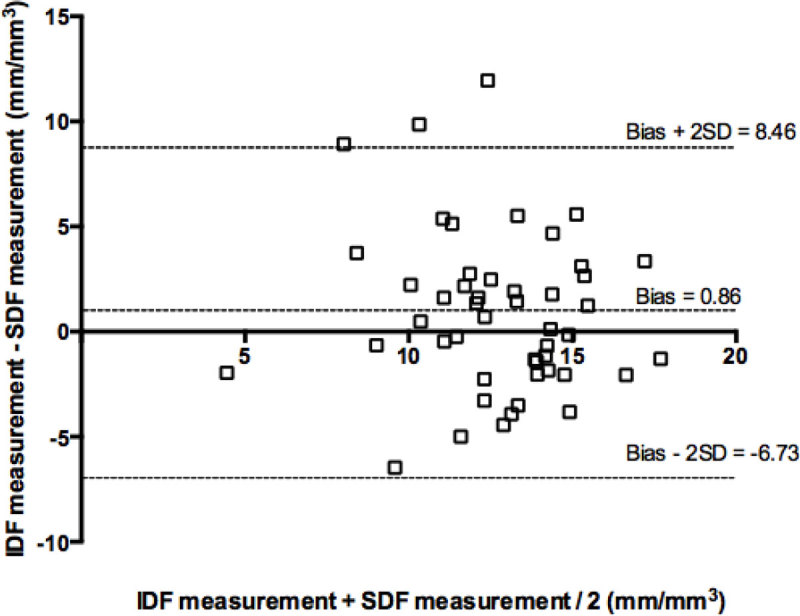
**Bland Altman plot of TVD measurements**

88 matched video sequences were obtained, 40 during baseline and 48 during the shocked state. There were no differences in density or flow data recorded from the two devices at baseline [TVD IDF 14.2 ± 2.4 / TVD SDF 13.2 ± 2.0, p 0.17] [MFI IDF 3 (2.8-3.0) / MFI SDF 3 (2.9-3.0), p 0.36] or during the shocked state [TVD IDF 11.64 ± 3.3 / TVD SDF 11.4 ± 4.0 p = 0.98] [MFI IDF 1.9 (0.6-2.7) / MFI SDF 1.7 (0.3-2.6) p 0.55]. Bland and Altman analysis (Figure 1) showed no evidence of significant bias. Vessel contrast was significantly better when using the IDF device for both capillaries [17.1 ± 3.9 (IDF) v 3.4 ± 3.6 (SDF), p = 0.0006] and venules [36.1 ± 11.4 (IDF) v 26.4 ± 7.1 (SDF) p 0.014]

## Conclusion

The Braedius Cytocam is a useful new device for assessing the shocked microcirculation with significant improvements in image contrast and quality over precursor devices. Data produced by the two devices appears comparable and this should aid investigators wishing to transition to the new device.

